# Practice of pharmaceutical services and prescription analysis in internet-based psychiatric hospitals during COVID-19 pandemic in Wuxi, China

**DOI:** 10.3389/fpsyt.2023.1195298

**Published:** 2023-07-20

**Authors:** Zhiqiang Du, Ying Jiang, Rongrong Lu, Qin Zhou, Yiting Pan, Yuan Shen, Haohao Zhu

**Affiliations:** Mental Health Center of Jiangnan University, Wuxi Central Rehabilitation Hospital, Wuxi, Jiangsu, China

**Keywords:** psychiatric hospital, internet, pharmaceutical services, prescription analysis, pandemic

## Abstract

**Objective:**

To study the practice of pharmaceutical services in internet-based psychiatric hospitals, and to analyze the prescriptions to ensure the safety and efficacy of internet-based medication in Wuxi, China.

**Methods:**

All 1,259 internet-based prescriptions from our hospital in 2022 were collected, and data on patients’ age, gender, diagnosis, medications used, medication types, dosage forms, rationality of medication use, and reasons for irrationality were analyzed through descriptive statistics.

**Results:**

In the electronic prescriptions of internet-based psychiatric hospitals, females accounted for the majority (64.50%), with a female-to-male ratio of 1.82:1. Middle-aged and young adults accounted for the majority of patients (57.50%). There were 47 diagnosed diseases involved, with 89 types of medications used and 1,938prescriptions issued. Among them, there were 78 types of western medicine with 1,876 prescriptions (96.80%), and 11 types of traditional Chinese medicine with 62 prescriptions (3.20%). The main medications used were anti-anxiety and antidepressant medications (44.94%) and psychiatric medications (42.21%). The dosage forms were all oral, with tablets (78.53%), capsules (17.54%), and solution preparations (2.17%) being the top three in frequency. According to the prescription review results, the initial pass rate of internet-based system review was 64.26%. After intervention by the internet-based system and manual review by pharmacist reviewers, the final pass rate of internet-based prescriptions reached 99.76%.

**Conclusion:**

The practice of pharmaceutical services and prescription analysis in internet-based psychiatric hospitals could significantly improve medication rationality, which fills the research gap in this field. In addition, it promotes the transformation of pharmaceutical service models.

## 1. Introduction

During the pandemic period of COVID-19, hospitals are both the first line of defense in pandemic prevention and control and a high-risk place for the spread of the pandemic (1–3). For patients with chronic mental illness, there is not only the risk of infection but also the difficulty of obtaining medication from hospitals, and even the danger of discontinuing medication (4, 5). According to the Notice on Strengthening the Treatment and Management of Severe Mental Disorders During the COVID-19 Pandemic issued by the National Health Commission of China (6), 323 cases of severe mental disorders have been diagnosed with COVID-19, and 43 cases were suspected of having COVID-19, involving 17 provinces across the country. Due to the high degree of specialization, most psychiatric hospitals are not equipped with internal medicine. Currently, COVID-19 related research mainly focuses on general hospitals, and there is less research on psychiatric pandemics.

To reduce the risk of crowd gathering and cross-infection in hospitals during the pandemic period, and to meet the needs of patients for safe, timely, and convenient access to medication, electronic medical systems have been introduced through several approaches. Anthony Jnr emphasized the important of telehealth based on a technological organizational environment framework in public health emergencies (7). Other studies also confirmed that the telemedicine and eHealth could protect both medical practitioners and patients, reduce the spread of COVID-19 (8, 9). Telepsychiatry has also proven as a promising way to improve the mental health assistance, however, it has not been underused (10).

The National Health Commission of China issued a notice requiring medical institutions nationwide to fully leverage the unique advantages of “Internet + medical care” to improve online drug supply guarantee services (11), that is, after the pharmacist reviews the common and chronic disease prescriptions issued online, medical institutions and drug business enterprises can entrust qualified third-party institutions for delivery, expand the space for online medical services, and alleviate the spread of the pandemic. During the pandemic, our hospital (Mental Health Center of Jiangnan University) responded actively, and opened a trial run of the online outpatient department of the psychiatric hospital on October 10, 2021, which was officially launched on January 1, 2022. This study provided an in-depth analysis of the pharmacy service practice and outpatient prescription situation of the internet hospital to improve the quality of pharmacy services in psychiatric internet hospitals, standardize the operation of internet hospitals during the pandemic.

## 2. Materials and methods

During the pandemic, the Internet hospital was established, and the internet electronic prescription could be obtained online. Through a retrospective analysis of the hospital information system, all 1,259 electronic outpatient prescriptions in 2022 were retrieved from the internet hospital of the psychiatric hospital in Wuxi, China. According to the prescription content, patients’ age, gender, online consultation disease category, prescription medication name, type, dosage form, and other information were recorded in an Excel table, and statistical analysis was conducted on the prescription situation, distribution of diseases, medications used by patients, type, dosage form, rationality of medication use, and reasons for irrationality. The evaluation of prescription medication use rationality was based on the “Prescription Management Measures” (original Ministry of Health document No. 53), the “Hospital Prescription Evaluation Management Norms (Trial)” (Health and Medical Administration Letter [2010] No. 28), drug instructions, published domestic and foreign literature, and published books, etc.

Descriptive statistics were used. Data was inputted to EpiData 3.1 software and statistical analyzed through SPSS 20.0. The counting data was described as the number of cases (composition ratio), and the measurement data was described by x ®±s.

## 3. Results

### 3.1. Basic information of electronic prescriptions

A total of 1,259 electronic prescriptions were collected, of which 812 cases (64.50%) were females, and 447 cases (35.50%) were males. The ratio of females to males was 1.82:1. Young adults aged 18–39 accounted for the largest proportion of patients, with 724 cases (57.50%). According to the “Hospital Prescription Review Management Specification,” the number of drug varieties contained in per prescription generally complied with the regulations. One drug was prescribed in the majority of cases, with 702 prescriptions (55.76%). Refer to Table 1.

**Table 1:** Basic situation of electronic prescriptions in the internet-based psychiatric hospital.

Basic information		Number of internet prescriptions	Proportion (%)
Gender	Female	812	64.50
	Male	447	35.50
Age	7–17	209	16.60
	18–39	724	57.50
	40–59	231	18.35
	≥60	95	7.55
Number of drug varieties per prescription (types)	1	702	55.76
	2	453	35.98
	3	89	7.07
	4	12	0.95
	5	3	0.24

### 3.2. Distribution of diseases

All the electronic prescriptions issued by our internet-based psychiatric hospital were psychiatric-related disorders and for follow-up treatments of non-emergency cases. Of the 1,259 electronic prescriptions, a total of 47 diseases were involved, and the top three diseases were anxiety depression (17.00%), anxiety disorder (14.38%) and depressive episode (10.88%) (Table 2).

**Table 2:** Distribution of the top 10 diseases.

Diseases	Number of internet prescriptions	Proportion (%)
Anxiety depression	214	17.00
Anxiety disorder	181	14.38
Depressive episode	137	10.88
Schizophrenia	136	10.80
Recurrent depressive disorder	65	5.16
Bipolar disorder	65	5.16
Mood disorder	57	4.53
Childhood emotional disorder	53	4.21
Obsessive–compulsive disorder	52	4.13
Nervous exhaustion	40	3.18

### 3.3. Frequency ranking of drug prescriptions

Of the 1,259 electronic prescriptions investigated in the internet-based psychiatric hospital, 89medications were involved, and the medications were not listed as narcotics, Class I or II psychotropic medications, high-risk or high-alert medications under special management by the government. The top 10 medications in terms of prescription frequency were quetiapine fumarate tablets (9.39%), escitalopram oxalate tablets (8.10%), olanzapine tablets (7.59%), duloxetine hydrochloride delayed-release capsules (7.33%), venlafaxine hydrochloride sustained-release capsules (4.64%), mirtazapine tablets (4.33%), fluvoxamine maleate tablets (4.23%), aripiprazole tablets (4.18%), sertraline hydrochloride tablets (4.08%), and agomelatine tablets (3.46%) (Table 3).

**Table 3:** The top 10 medications in terms of frequency of electronic prescription.

Medications	Frequency	Proportion (%)
Quetiapine Fumarate tablets	182	9.39
Escitalopram Oxalate tablets	157	8.10
Olanzapine tablets	147	7.59
Duloxetine Hydrochloride Gastro-resistant capsules	142	7.33
Venlafaxine Hydrochloride Sustained-release capsules	90	4.64
Mirtazapine tablets	84	4.33
Fluvoxamine Maleate tablets	82	4.23
Alprazolam tablets	81	4.18
Sertraline Hydrochloride tablets	79	4.08
Agomelatine tablets	67	3.46

### 3.4. Distribution of medication types

In the medication variety of electronic prescriptions issued by internet-based mental health hospitals surveyed in this study, a total of 78 Western medicines were involved with a prescription frequency of 1876 times (96.80%), and 11 traditional Chinese medicines were involved with a prescription frequency of 62 times (3.20%). Mainly, anti-anxiety and antidepressant medications accounted for 871 (44.94%), and psychotropic medications accounted for 818 (42.21%), as shown in Table 4.

**Table 4:** Distribution of medication types.

Types	Frequency	Proportion (%)
Western medicines	1876	96.80
Anti-anxiety and antidepressant medications	871	44.94
Psychotropic medications	818	42.21
Antiepileptic medications	104	5.37
Anti-dementia medications	21	1.08
Antiplatelet medications	12	0.62
Vasodilator medications	9	0.46
Anticholinergic medications	9	0.46
Laxatives	7	0.36
Anti-anemia medications	5	0.26
Antidiabetic medications	5	0.26
Antihypertensive medications	4	0.21
Vitamins	3	0.15
Hepatoprotective medications	3	0.15
Cardiovascular medications	2	0.10
Antibacterial medications	2	0.10
Thyroid hormone medications	1	0.05
Chinese medicines	62	3.20
Tranquillization and sleep-promoting	55	2.84
Blood-nourishing and qi-regulating	3	0.15
Promoting blood circulation and removing blood stasis	3	0.15
Liver-clearing and gallbladder-benefiting	1	0.05

### 3.5. Distribution of medication dosage forms

The medication formulations prescribed by the online psychiatric hospital were all oral formulations, with tablets accounting for the top three at 1522 times (78.53%), followed by capsules at 340 times (17.54%), and liquid formulations at 42 times (2.17%) (Table 5).

**Table 5:** Distribution of medication dosage forms.

Dosage forms	Frequency	Proportion (%)
Tablets	1,522	78.53
Capsules	340	17.54
Liquid formulations	42	2.17
Granules	26	1.34
Suppositories	7	0.36
Pills	1	0.05
Total	1938	100.00

### 3.6. Analysis of irrational medication use

Among the 1,259 electronic prescriptions issued, 450 (35.74%) were judged to be irrational by the internet system. The types of irrational medication use included 254 cases (20.17%) of overindication, 130 cases (10.33%) of special population medication problems, 60 cases (4.77%) of drug interactions, and 6 cases (0.48%) of inappropriate dosage and administration. After initial review by the internet prescription review system and pharmacist intervention, and confirmation by the doctor’s signature, a total of 3 internet prescriptions were deemed irrational after final review by the pharmacist, accounting for 0.24% of the total prescriptions. The main problems were drug interactions in 2 cases (0.16%) and overindication in 1 case (0.08%) (Table 6).

**Table 6:** Irrational medication use situation.

Types	Judged by internet system	Proportion (%)	Judged by pharmacist	Proportion (%)
Overindication	254	20.17	1	0.08
Special populations	130	10.33	0	0.00
Drug interaction	60	4.77	2	0.16
Inappropriate dosage and administration	6	0.48	0	0.00
Total	450	35.74	3	0.24

## 4. Discussion

This study found that during the pandemic in Wuxi, China, female patients accounted for the majority (64.50%) of electronic prescriptions in the online psychiatric hospital, with a female-to-male ratio of 1.82:1. Previous studies have shown that women were more likely than men to experience negative emotions (12, 13) and have a significantly higher proportion of mental illnesses such as depression, recurrent depressive disorders, and anxiety disorders (14). This study found that young adults aged 18–39 were the main age group of patients (57.50%). Previous research has shown that the number of cases of mental depression in young adults was the highest (15, 16), and young adults consult online more frequently than older people and find it more convenient. According to the “Hospital Prescription Review and Management Standards,” the number of medication types prescribed in electronic prescriptions for psychiatric hospitals in this study generally complied with regulations, with one medication type accounting for the majority (55.76%).

Our online psychiatric hospital mainly provides follow-up and chronic disease prescription services for non-emergency or non-first-time patients with mental illnesses. This study found that our hospital’s electronic prescriptions covered a total of 47 types of diseases, with the top three being anxiety depression (17.00%), anxiety disorders (14.38%), and depressive episodes (10.88%). Previous studies have shown that sudden public health emergencies can lead to changes in social and economic conditions and lifestyle, which can lead to varying degrees of psychological problems such as depression and anxiety (17–19). During the COVID-19 pandemic, measures such as work stoppages, school closures, and quarantine of residential areas were taken to control the spread of the virus. Due to medical isolation or home quarantine, the daily routines were disrupted, and they were worried about the impact on work or family during isolation or illness, leading to significant psychological pressure and obvious depression and anxiety (19). Patients with mental illnesses are more susceptible to illness, and online psychiatric hospitals can help reduce the risk of cross-infection from offline consultations, facilitate consultations and medication management, and provide medication guidance.

The medication catalog should meet the common diseases and chronic disease treatment needs of online psychiatric hospitals. Medications with high individualized risk and requiring personalized medication guidance should not be included in the catalog (20). This study surveyed a total of 1,259 electronic prescriptions in the online psychiatric hospital, involving 89 types of medications with a prescription frequency of 1,938 times. Among them, there were 78 types of Western medicine with a prescription frequency of 1,876 times (96.80%), and 11 types of traditional Chinese medicine with a prescription frequency of 62 times (3.20%). The prescriptions did not involve narcotic medications, Class I and Class II psychotropic medications, and high-risk or high-danger medications that are subject to special management regulations by the government. The majority of these medications are anti-anxiety and anti-depressant medications (44.94%) and psychiatric medications (42.21%), which indicated the influence of COVID-19 on the mental health (18, 19).

This study found that all the psychiatric medications prescribed by internet-based hospitals were oral medications. As a psychiatric hospital, the medications used in outpatient services are mainly oral psychiatric medications, with a low use of injectable medications. Additionally, medications with high distribution requirements, such as injectable medications and cold chain storage medications, have not yet been included in the online medication delivery catalog and are currently only available for patients to obtain at the hospital.

This study found that the initial qualification rate of prescriptions reviewed by the Internet system was 64.26%, but after the Internet system intervened and the reviewing pharmacist conducted manual review, the final qualification rate of the internet prescription reached 99.76%. The types of unreasonable medication use mainly determined by the initial review of our Internet system include off-label use (20.17%) and special population medication use issues (10.33%). For the off-label use of medications, special population medication use, medication interactions, improper use and dosage, etc. in the Internet psychiatric hospital, corresponding measures can be taken to establish a feasible Internet electronic prescription review system, strictly follow the relevant regulations, and ensure the accuracy and effectiveness of each electronic prescription. During the process of issuing electronic prescriptions, the Internet prescription review system should be continuously updated and maintained (21, 22). If there are issues such as off-label use, medication dosage, medications not suitable for the symptoms, or drug interactions, an alert should be issued to reduce the occurrence of unreasonable prescriptions (23–25).

Although the study pointed the importance of internet hospital during the pandemic, the study was a single-center study, the sample size was small and the types of involved medication was limited. Therefore, the model needs to be continuously improving, in order to enhance its clinical applicability.

## 5. Conclusion

The internet psychiatric hospital has changed the traditional medical process for patients during the pandemic period, which could significantly improve medication rationality. This study analyzed the pharmacy services and electronic prescription situation of the online psychiatric hospital during the pandemic, as well as the application characteristics of medication varieties, types, dosage forms, and usage, which filled the gap in domestic and foreign research, providing reference for further studies. On the other hand, it can improve the quality of pharmacy services in the online psychiatric hospital. Meanwhile, follow-up pharmacy services can be extended to include online follow-up visits and monitoring of adverse drug reactions by clinical pharmacists for patients who receive medication online. Especially during this special period of nationwide unity and overcoming difficulties, pharmaceutical personnel should integrate pharmacy services with new technologies and concepts on the Internet while taking proper protective measures, actively promoting the transformation of pharmacy service models, providing new ideas for pharmaceutical workers in the fight against the pandemic, and contributing to the overall victory of the pandemic prevention and control.

## Data availability statement

The original contributions presented in the study are included in the article/supplementary material, further inquiries can be directed to the corresponding authors.

## Ethics statement

The studies involving human participants were reviewed and approved by Wuxi Mental Health Center. Written informed consent for participation was not required for this study in accordance with the national legislation and the institutional requirements.

## Author contributions

ZD and HZ conceived the study. ZD, YJ, RL, YP, and QZ collected the report. YJ and ZD wrote the manuscript. YS and HZ edited the manuscript. All authors contributed to the article and approved the submitted version.

## Funding

The work is supported by the National Natural Science Foundation of China (no. 82104244), Wuxi Municipal Health Commission (no. Q202101 and ZH202110), Wuxi Taihu Talent Project (no. WXTTP2020008 and WXTTP2021), Wuxi Medical Development Discipline Project (no. FZXK2021012), and Jiangsu Research Hospital Association for Precision Medication (JY202105).

## Conflict of interest

The authors declare that the research was conducted in the absence of any commercial or financial relationships that could be construed as a potential conflict of interest.

## Publisher’s note

All claims expressed in this article are solely those of the authors and do not necessarily represent those of their affiliated organizations, or those of the publisher, the editors and the reviewers. Any product that may be evaluated in this article, or claim that may be made by its manufacturer, is not guaranteed or endorsed by the publisher.
